# Prevalence of IVIG resistance in Kawasaki disease: a systematic review and meta-analysis

**DOI:** 10.3389/fped.2025.1566590

**Published:** 2025-07-04

**Authors:** Shuting Zou, Bingyu Hu

**Affiliations:** Department of Pediatrics, The First People’s Hospital of Jiashan, Jiashan County, Jiaxing, Zhejiang, China

**Keywords:** Kawasaki disease, IVIG resistance, prevalence, risk factors, randomized controlled trials, clinical outcomes

## Abstract

**Background:**

Kawasaki Disease (KD) is an acute vasculitis primarily affecting children, with intravenous immunoglobulin (IVIG) being the standard treatment and leading to increased risk of coronary artery abnormalities.

**Objective:**

This systematic review and meta-analysis aim to evaluate the prevalence of IVIG resistance in KD and identify potential predictors and outcomes associated with this resistance.

**Methods:**

A comprehensive search of PubMed, Medline, Embase, and other relevant databases was conducted to identify studies reporting IVIG resistance in KD patients. Data on prevalence rates, patient demographics, and associated factors were extracted and analyzed.

**Results:**

The analysis included 26 studies with a total of 46,461 patients. The overall prevalence of IVIG resistance was found to be 14% (95% CI: 12%–16%), and The prevalence among males and females was 9% (95% CI; 7% to 10%) and 4% (95% CI; 3.7% to 4.3%), respectively.

**Conclusion:**

IVIG resistance remains a significant challenge in the management of KD. Identifying patients at higher risk for IVIG resistance and developing alternative treatment strategies are crucial for improving outcomes in this population.

## Introduction

Kawasaki Disease (KD) is an acute systemic vasculitis primarily affecting children under the age of five. It is the leading cause of acquired heart disease in children, with coronary artery lesions (CALs) being the most common and life-threatening complication. If left untreated, CALs can lead to severe outcomes such as coronary artery dilatation, aneurysm formation, lumen stenosis, occlusion, and potentially fatal myocardial infarction (1).

KD triggers the release of various inflammatory factors, resulting in a cascade amplification effect. Although KD is a self-limited inflammatory process, it can become life-threatening depending on the extent of cardiac involvement. Diagnosis is based on clinical criteria, including fever, exanthema, conjunctivitis, changes in extremities, erythema of oral mucosa and lips, and cervical lymphadenopathy. Early recognition and treatment are crucial to lower the risk of cardiac complications, the etiology of KD remains unknown, though clinical, laboratory, and epidemiological features suggest an infectious origin or trigger. Immune system activation is a notable feature of KD, with elevated concentrations of proinflammatory cytokines and chemokines being studied for their potential to improve future anti-inflammatory therapies (2).

The standard treatment for KD involves high-dose intravenous immunoglobulin (IVIG) at 2 g/kg combined with aspirin, reducing the incidence of CALs from 20%–25% to 2%–4%. However, initial IVIG treatment fails in 7.5%–26.8% of KD patients, who continue to experience inflammatory reactions and remain at risk for CALs. Severe complications such as Kawasaki disease shock syndrome or macrophage activation syndrome can occur, posing significant threats to patient survival. Thus, in-depth analysis of factors influencing IVIG insensitivity, early prediction, and timely intervention are critical to reducing cardiac damage in IVIG-resistant KD (1). The American Heart Association (AHA) recommends early administration of IVIG to significantly reduce CAL incidence. Yet, up to 20% of KD patients exhibit IVIG resistance, strongly associated with CAL occurrence. Recent literature has focused on identifying predictors for IVIG resistance, aiming to implement additional therapeutic measures to reduce CAL incidence through early diagnosis. Research indicates various predictors, including male sex, high C-reactive protein (CRP) levels, decreased platelet counts, and elevated neutrophil ratios (3).

Therefore, early identification of IVIG resistance in KD patients is vital for initiating intensive treatments such as corticosteroids, potentially benefiting these patients by reducing the risk of severe cardiac complications (4).

This systematic review and meta-analysis seek to determine the prevalence of IVIG resistance in KD, assess the associated risk factors, and provide insights into the potential for early prediction and intervention. Understanding these elements is vital for optimizing treatment strategies and improving the prognosis for children affected by this complex disease.

## Method

This systematic review and meta-analysis followed the Preferred Reporting Items for Systematic Reviews and Meta-Analyses (PRISMA) guidelines.

### Search strategy

A comprehensive search was conducted in the following electronic databases: PubMed, Cochrane Library, Embase, Web of Science, Google Scholar, Semantic Scholar, and ResearchRabbit from January 2008 to October 31, 2024. The search terms (Mesh) included (“Kawasaki Disease” AND “IVIG resistance”) AND (prevalence OR incidence). Additional relevant articles were obtained by searching the reference lists of the articles. The search was restricted to articles published in English.

### Inclusion criteria (PIOS)

**Population:** Children diagnosed with Kawasaki disease.

**Intervention:** Initial treatment with intravenous immunoglobulin (IVIG).

**Outcomes:** Studies reporting the prevalence of IVIG resistance.

**Study Design:** Cohort studies published in peer-reviewed journals.

### Exclusion criteria

Studies were excluded from the review if they were non-original research (e.g., case reports), lacked sufficient data on IVIG resistance prevalence, involved non-cohort designs, included adult populations or mixed-age groups without separate pediatric data, used treatments other than standard-dose IVIG, were not published in English, or represented duplicate data from the same patient population.

### Data extraction

A standardized data extraction form was developed to systematically collect relevant information from each included study. Extracted data included study characteristics such as first author, publication year, sample size, participant demographics (including age), Kawasaki disease type, laboratory parameters, and reported prevalence of IVIG resistance.

### Statistical analysis

The meta-analysis was conducted using STATA Ver. 17 and the Dichotomous outcomes were analyzed using risk ratios (OR) with 95% confidence intervals (CI), and continuous outcomes were analyzed using standardized mean differences (SMD) with 95% CI.

The random or fixed effect models were used to pool the data. The Q test and *I*^2^ index were used to assess the heterogeneity of the studies. If the *I*^2^ value was greater than 50%, the heterogeneity was considered substantial, and the sources of heterogeneity were explored (5). The random or fixed effect models were used to pool the data. A funnel plot was used to assess the publication bias. Meta-regression and the Eger test were used to explore the significance of publication bias (6).

## Results

**Figure 1:**
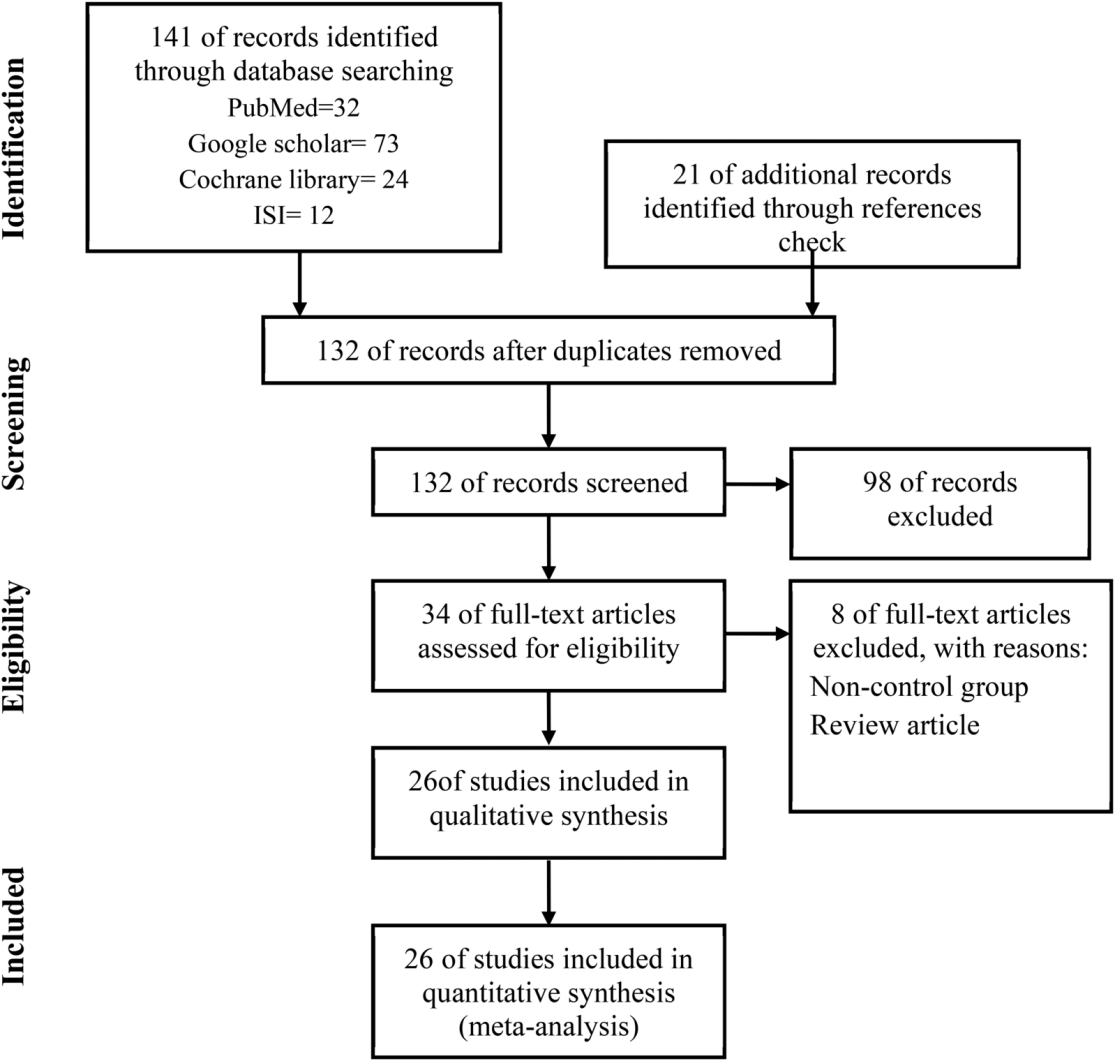
The PRISMA flow diagram includes studies.

141 related studies were found by searching in the databases, 32 studies in PubMed, 73 in Google Scholar, 24 studies in Cochrane, and 12 studies in Web of Sciences. 21 studies were found by checking the references, after removing 30 duplicate studies, 132 articles were screened and reviewed. Out of 132 studies, 98 studies were excluded based on non-relevant titles or abstracts, 8 studies were excluded for the following reasons:

No original research, insufficient data, and no relevant outcomes. A total of 26 articles met our inclusion criteria and were finally included in this meta-analysis (Figure 1).

### Characteristics of included studies

In 26 studies, the sample size was 46,461 people. While most studies included both complete and incomplete KD, a few explicitly reported data on atypical KD forms, which appear to be associated with a similar or potentially higher risk of IVIG resistance (4, 12). The general characteristics of the studies included in the meta-analysis are given in Table 1.

**Table 1:** Characteristics of included studies.

Author	Year	Sample size	Age (month)	Total of IVIG-resistant	Male/female	Key laboratory parameters
Wang et al. (1)	2023	177	33.00	21	17/4	WBC ↑, Neutrophil-Lymphocyte Ratio (N/L ratio) ↑, Hematocrit (HCT) ↓, Albumin (ALB) ↓, Total Bilirubin (TBIL) ↑, Lactate Dehydrogenase (LDH) ↑, Creatinine (Cr) ↑
Liu et al. (7)	2021	831	55.20	118	61/57	Systemic Immune-Inflammation Index (SII) ↑, Neutrophil-to-Lymphocyte Ratio (NLR) ↑, Platelet-to-Lymphocyte Ratio (PLR) ↑ (SII predictive but less accurate than NLR; SII not reliable if thrombocytopenia present)
Zheng et al. (3)	2021	30,312	-	4,750	–	N/A
Zhou et al. (8)	2023	108	33.60	31	–	ESR ↑ (≥79.5 mm/h), BECN1 ↓ (≤0.645), LC3II ↓ (≤0.481), ALT ↑, AST ↑, GLB ↑, r-GT ↑, IgG ↑, PCT ↑, ESR ↑, RBC ↓, HGB ↓, ALB ↓, A/G ↓, CK ↓, ATG16L1 ↓
Wu et al. (9)	2020	277	22.00	31	24/7	Neutrophil count ↑, Lymphocyte count ↓, Mean Platelet Volume (MPV) ↑, Serum albumin ↓
Cui et al. (4)	2024	962	38.19	138	83/55	Fibrinogen **↑** Albumin **↓**
Liu et al. (10)	2023	153	7.00	41	30/11	Before IVIG: Total bilirubin/Albumin ratio (B/A) ↑, Albumin ↓, Sodium ↓, Neutrophil-to-lymphocyte ratio (NLR) ↑, Platelet-to-lymphocyte ratio (PLR) ↑, Prognostic Nutritional Index (PNI) ↓ After IVIG: Capillary Leakage Index (CLI) ↑, Systemic Immune-inflammation Index (SII) ↑, WBC ↑, Neutrophils ↑, CRP ↑, ALT ↑, Albumin ↓
Lim et al. (11)	2024	143	7.07	45	31/14	Total bilirubin ↑, Albumin ↓
Zhang et al. (12)	2023	907	32.50	66	38/28	Albumin ↓, Neutrophils % ↑, CRP ↑, CRP/Albumin ratio ↑, Lymphocytes % ↓
Zhang et al. (13)	2024	649	33.00	76	52/24	LDL-C ↓, HDL-C ↓, TC ↓, CRP ↑, Neutrophils ↑, PLT ↓
Kong et al. (14)	2019	300	25.00	29	-	– ↑ D-dimer (cutoff: ≥1.09 mg/L, sensitivity 87%, specificity 56.3%) – ↑ Globulin (cutoff: ≥34.7 g/L, sensitivity 62.1%, specificity 82.3%) – ↑ Serum ferritin (cutoff: ≥269.7 ng/ml, sensitivity 42.9%, specificity 88.8%)
Chantasiriwan et al. (15)	2018	217	17.00	26	22/4	– ↓ Hematocrit (≤ 30%) – ↓ Platelet count (≤300 × 10⁹/L) – ↑ Neutrophil-to-lymphocyte ratio (≥3.2) – ↑ AST (≥40 U/L)
Kaya Akca et al. (16)	2021	129	44.60	16	10/6	– ↓ Platelet count (<300 × 10⁹/L) – ↑ Serum gamma-glutamyl transferase (GGT) levels
Lee et al. (17)	2014	91	3.80	11	6/5	– Before IVIG treatment: • ↑ White blood cell count (WBC) • ↑ % Neutrophils • ↑ ESR • ↑ CRP • ↓ Sodium • ↑ CK and CK-MB • ↑ NT-proBNP – After IVIG treatment (24–36 h): • ↓ Hemoglobin • ↑ WBC • ↑ % Neutrophils • ↓ % Lymphocytes • ↑ CRP, CK, CK-MB, NT-proBNP
Fury et al. (18)	2011	20	1.85	8	5/3	– ↑ Transcript abundance of IL-1 pathway genes: • IL-1 receptor • Interleukin receptor associated kinase • p38 mitogen-activated protein kinase (MAPK) – ↑ MMP-8 (matrix metalloproteinase-8)
Fu et al. (19)	2013	1,177	47.00	211	144/67	– Increased percentage of neutrophils – Elevated C-reactive protein (CRP) – Low albumin – Elevated total bilirubin – Clinical signs: polymorphous exanthema, changes around anus, days of illness at initial treatment
Sato et al. (20)	2013	105	41.50	21	11/10	– Elevated Interleukin-6 (IL-6) (≥140 pg/ml high risk; 70–140 pg/ml moderate risk) – High neutrophil percentage (≥75%) – Elevated C-reactive protein (CRP)
Yang et al. (21)	2019	1,360	1.75	78	50/28	– C reactive protein (CRP) ↑ ≥ 90 mg/L – Neutrophil percentage ↑ ≥ 70% – Sodium ion concentration ↓ < 135 mmol/L – Albumin ↓ < 35 g/L – Total bilirubin ↑ > 20 *μ*mol/L
Sánchez-Manubens et al. (2)	2016	399	44.70	67	44/23	– Egami score (≥3) showed low sensitivity (26%) and moderate specificity (82%) in this Western Mediterranean (Catalan) population – Egami score not a reliable predictor in this population
Zeng et al. (22)	2023	763	24.00	60	39/21	– ↑ Neutrophilic granulocyte percentage (NE%) ≥ 72.3% – Higher NE% independently associated with IVIG resistance
Kim et al. (23)	2016	703	33.31	118	62/56	– ↑ Total bilirubin – ↓ Platelet count – ↑ Neutrophil proportion
Kim et al. (24)	2018	5,151	33.04	524	317/181	– ↑ PMN (%) (Polymorphonuclear neutrophils) – ↑ NT-proBNP – ↑ CRP (Only independent predictor of CAL) – ↑ AST, ↑ ALT
Kim et al. (25)	2013	135	32.76	22	16/6	– NT-proBNP ≥ 1,093 pg/ml (Optimal sensitivity: 70.0%, specificity: 76.5%)
Park et al. (26)	2013	309	27.50	30	19/11	– ALT ≥ 84 IU/L – Total bilirubin ≥ 0.9 mg/dl – Also noted (but not independent predictors): ↑ AST, ↑ neutrophils, ↑ NT-proBNP, sterile pyuria
Bar-Meir et al. (27)	2017	312	29.00	42	26/16	N/A
Yi et al. (28)	2024	771	33.60	86	48/38	Systemic Immune Inflammation Index (SII) ↑, Systemic Inflammatory Response Index (SIRI) ↑, Pan-Immune Inflammation Value (PIV) ↑

WBC, white blood cell count; N/L ratio, neutrophil-lymphocyte ratio: ratio of neutrophils to lymphocytes; HCT, hematocrit: percentage of red blood cells in blood; ALB, albumin: serum albumin level; TBIL, total Bilirubin: total bilirubin level in blood; LDH, lactate dehydrogenase: enzyme involved in energy production; Cr, creatinine: kidney function marker; SII, systemic immune-inflammation index: composite index based on neutrophils, lymphocytes, and platelets; NLR, neutrophil-to-lymphocyte ratio: ratio of neutrophils to lymphocytes; PLR, platelet-to-lymphocyte ratio: ratio of platelets to lymphocytes; ESR, erythrocyte sedimentation rate: rate at which red blood cells sediment, indicating inflammation; BECN1, beclin-1 (a protein involved in autophagy), LC3II: microtubule-associated protein 1A/1B-light chain 3 (autophagy marker); ALT, alanine aminotransferase: liver enzyme; AST, aspartate aminotransferase: liver enzyme; GLB, globulin: serum globulin protein level; r-GT, gamma-glutamyl transferase: liver enzyme; IgG, immunoglobulin G: antibody level; PCT, procalcitonin: marker of bacterial infection; RBC, red blood cell count; HGB, hemoglobin: oxygen-carrying protein in red blood cells; A/G ratio, albumin to globulin ratio; CK, creatine kinase: muscle enzyme; ATG16L1, autophagy-related 16-like 1: protein related to autophagy; MPV, mean platelet volume: average size of platelets, fibrinogen: blood clotting protein, sodium (Na): serum sodium level; PNI, prognostic nutritional index: index reflecting nutrition and immune status; CLI, capillary leakage index: marker of vascular permeability; CRP, C-reactive protein: inflammatory marker; LDL-C, low-density lipoprotein cholesterol; HDL-C, high-density lipoprotein cholesterol; TC, total cholesterol, D-dimer: fibrin degradation product, marker of coagulation, serum ferritin: iron storage protein, acute phase reactant; PLT, platelet count, serum GGT, gamma-glutamyl transferase; CK-MB, creatine kinase-MB: cardiac muscle enzyme; NT-proBNP, N-terminal pro-brain natriuretic peptide: cardiac stress marker; IL-1, interleukin-1 pathway genes; MMP-8, matrix metalloproteinase-8: enzyme involved in tissue remodeling, polymorphous exanthema: rash seen in KD; CLI, capillary leakage index: a measure of capillary permeability; NE%, neutrophilic granulocyte percentage: percentage of neutrophils among white blood cells; PMN, polymorphonuclear neutrophils: a type of neutrophil; SIRI, systemic inflammatory response index: index involving neutrophils, monocytes, and lymphocytes; PIV, pan-immune inflammation value: composite immune-inflammation index; N/A, not applicable.

## Outcome

### Prevalence rates

**Figure 2:**
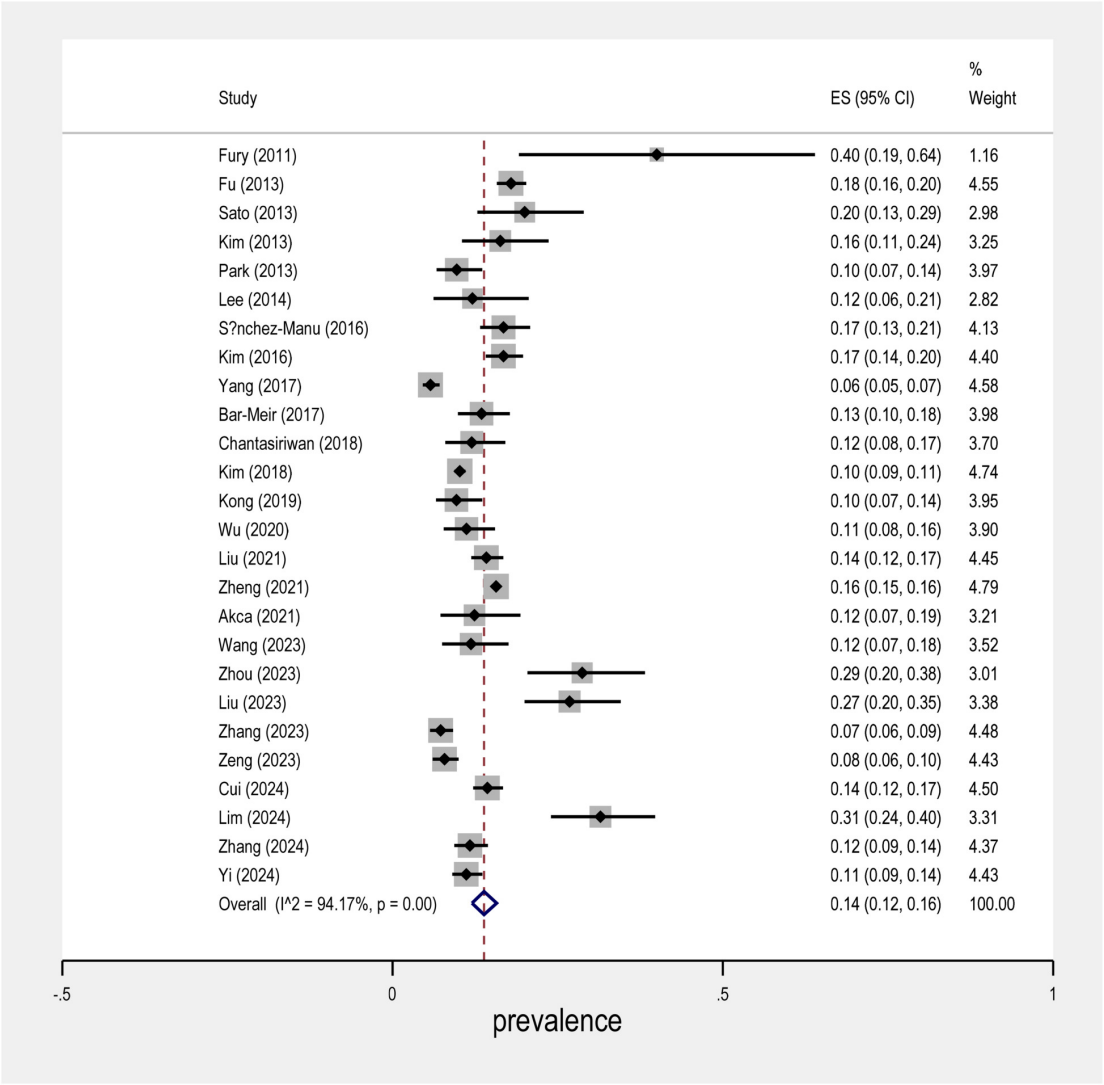
Forest plot of prevalence IVIG resistance in KD disease.

**Figure 3:**
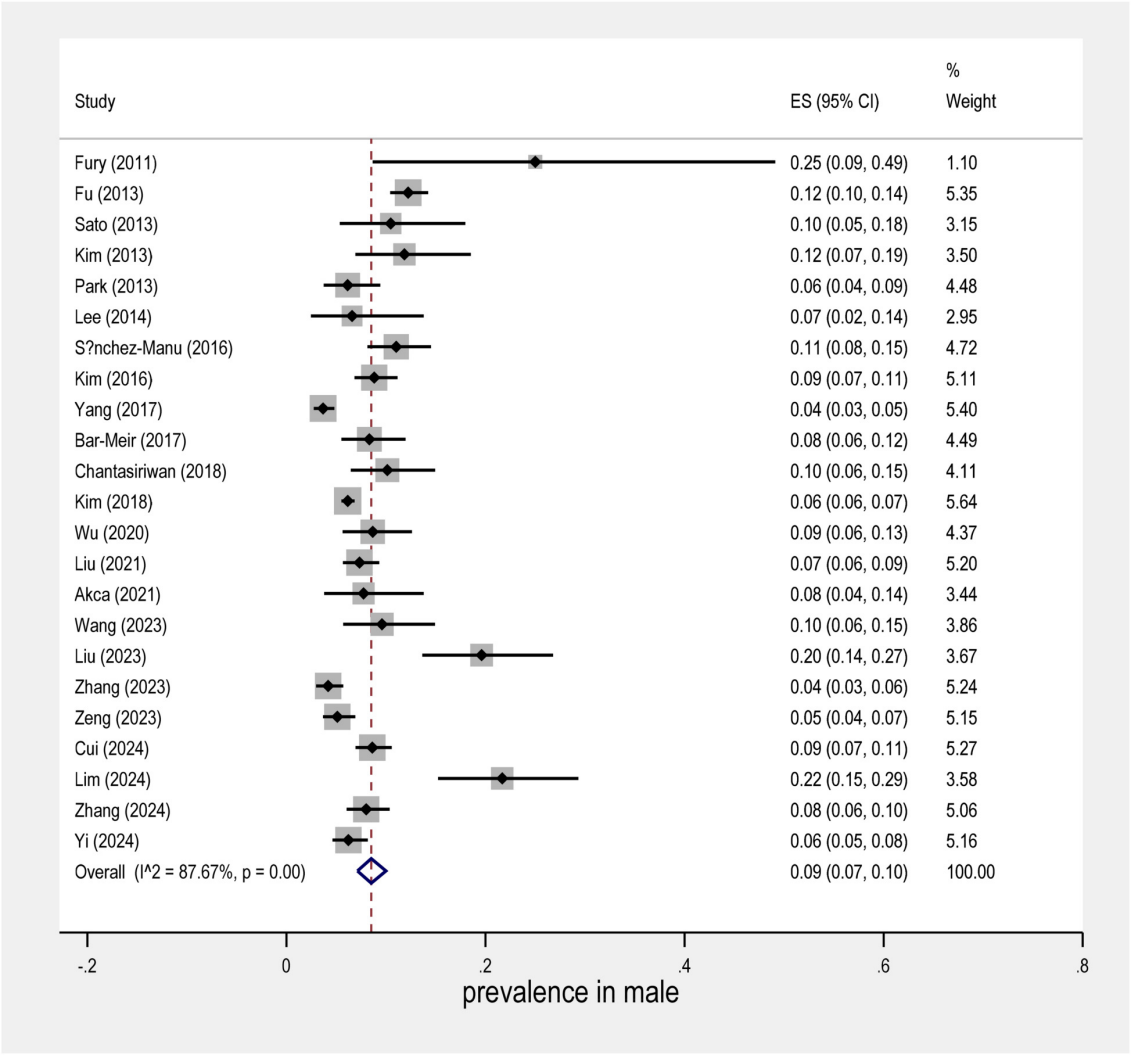
Forest plot of prevalence in males in the IVIG resistance group.

**Figure 4:**
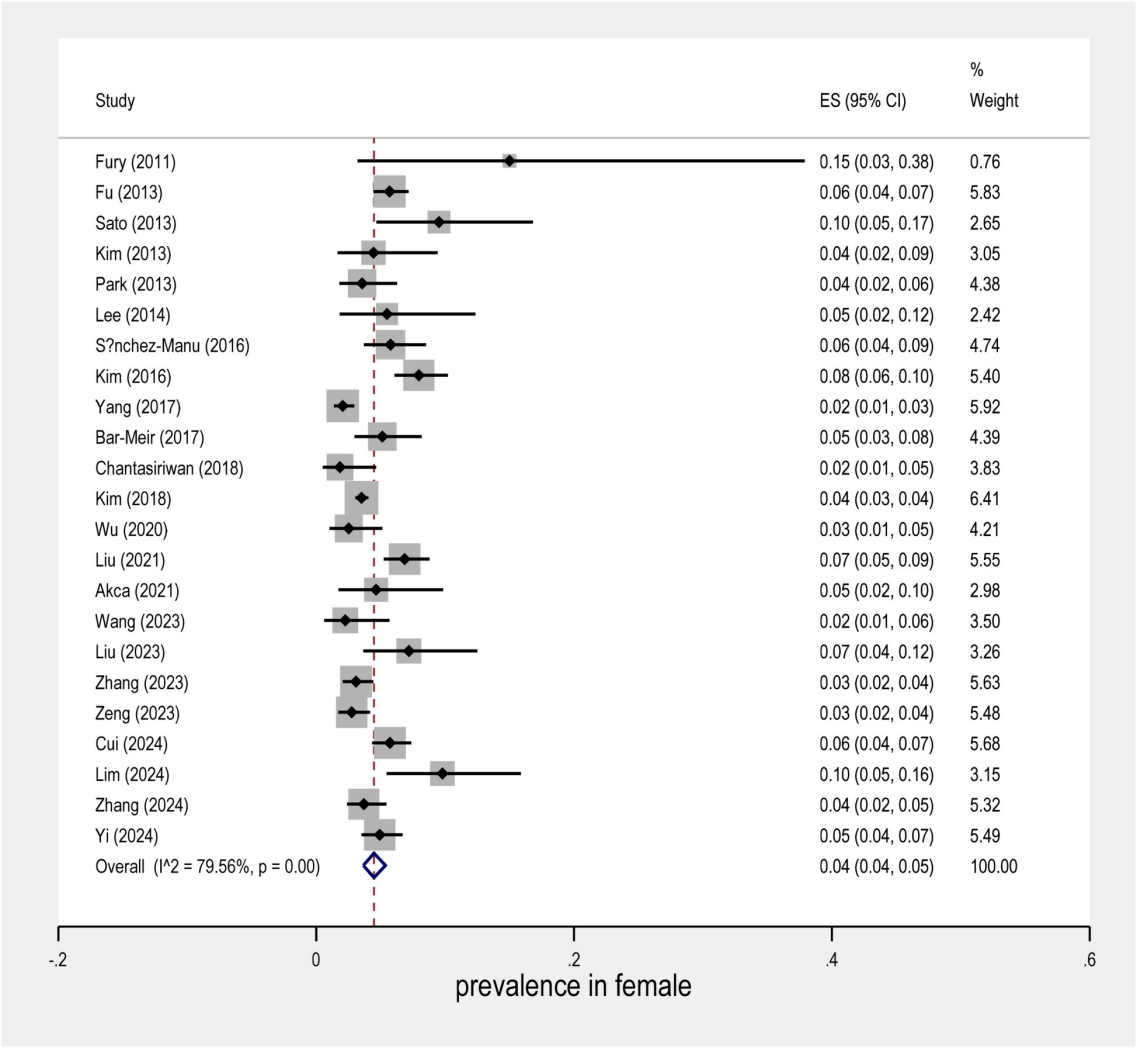
Forest plot of prevalence in females in the IVIG resistance group.

The meta-analysis conducted on the prevalence of intravenous immunoglobulin (IVIG) resistance in patients with Kawasaki Disease (KD) yielded important findings. The overall prevalence of IVIG resistance was found to be approximately 14%, with a 95% confidence interval (CI) ranging from 12% to 16% (Figure 2). This suggests that roughly one in seven KD patients may experience resistance to IVIG treatment. When examining the data by sex, it was observed that males had a prevalence rate of 9% (95% CI: 7% to 10%), as shown in Figure 3, while females exhibited a lower prevalence rate of 4% (95% CI: 4% to 5%) in Figure 4.

In terms of geographical variability, the study by Yang et al. reported the lowest prevalence of IVIG resistance, at only 6% (95% CI: 5% to 5%), suggesting that the occurrence of resistance may vary across different populations and clinical settings. In contrast, the study by Fury et al. showed a much higher rate of IVIG resistance, reaching over 40%. This marked difference highlights the influence of regional factors and the need for further investigation into the underlying causes of such disparities.

Further research is needed to fully understand the mechanisms behind IVIG resistance in KD and how best to tailor treatments to individual patients based on their risk profiles.

Overall, this meta-analysis underscores the complex nature of IVIG resistance in KD and calls for continued investigation into its prevalence, risk factors, and potential interventions.

**Risk Factors and Predictors** Across studies, predictors included:

High CRP and neutrophil ratio
Low albumin and platelet count
Elevated liver enzymes and bilirubin
Use of predictive scores (e.g., Egami, Kobayashi) with varied geographic accuracy.

### Publication bias

**Figure 5:**
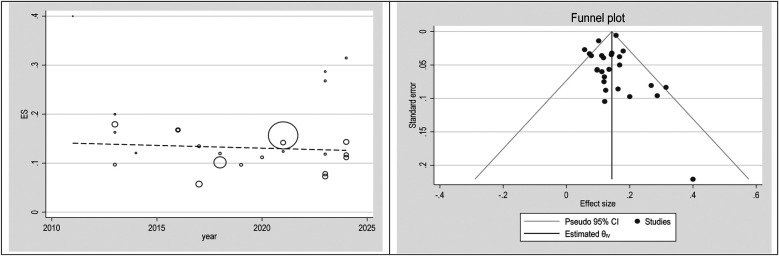
Bubble plot and funnel plot in total population.

**Figure 6:**
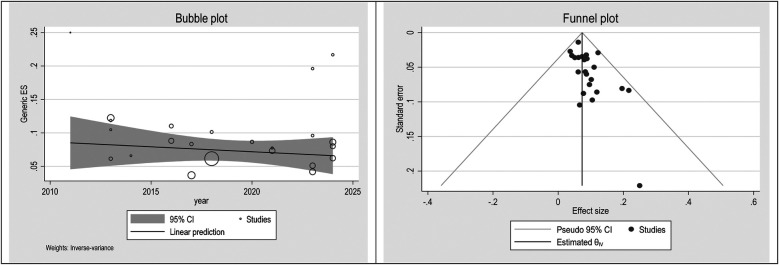
Bubble plot and funnel plot in male.

**Figure 7:**
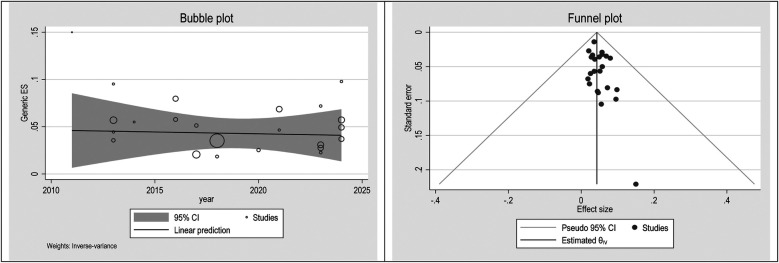
Bubble plot and funnel plot in females.

To check publication bias and small study, an effect funnel plot was used it demonstrated that the standard error of the majority of studies was low so the precision of included studies was high. bubble plot helps summarize the data in a way that highlights the relationships between multiple variables, the distribution of those relationships, and potential areas for deeper analysis. Figures 5–7 showed the effect of publication bias was not significant.

To explore source of heterogeneity and effect of year of publication on effect size multivariate meta-regression was applied, the results of meta regression showed that there were not significant association between year of publication and effects size (Table 2).

**Table 2:** Multivariate meta-regression: year and effects sizes.

Population group	Covariates	Coefficient	Std. err	t	*P*
In total	year	−0.0011287	0.0029123	−0.39	0.702
In male	year	−0.001471	0.0023117	−0.64	0.531
In female	year	−0.0003867	0.0023117	−0.17	0.867

## Discussion

The findings of this systematic review and meta-analysis provide a comprehensive understanding of the prevalence of intravenous immunoglobulin (IVIG) resistance in Kawasaki Disease (KD), a critical challenge in managing this pediatric vasculitis. Our analysis indicates that a significant proportion of KD patients do not respond to initial IVIG treatment, with prevalence rates ranging from 7.5% to 26.8% across various studies. This variability underscores the complexity of IVIG resistance and the need for tailored approaches in clinical practice. The high prevalence of IVIG resistance observed in KD patients has significant implications for clinical management. As CALs are the most severe complication associated with KD, timely and effective treatment is crucial to prevent long-term cardiac sequelae. Our findings align with previous studies that highlight the importance of early diagnosis and intervention to mitigate the risk of CALs (29, 30). Furthermore, understanding the factors contributing to IVIG resistance can guide the development of predictive models and personalized treatment strategies. Recent research has identified several predictors of IVIG resistance, including male gender, elevated C-reactive protein (CRP) levels, decreased platelet counts, and higher neutrophil ratios (31). These predictors can help clinicians identify patients at higher risk of resistance and consider alternative or adjunctive therapies, such as corticosteroids, in conjunction with IVIG (32).

Some studies also differentiated between complete, incomplete, and atypical KD, all of which are associated with IVIG resistance (4, 12). Several validated scoring systems have been developed to predict IVIG resistance in KD patients, including the Egami, Kobayashi, and Sano scores. These scores incorporate clinical and laboratory variables such as age, ALT levels, CRP, and neutrophil counts to stratify patients by risk. For example, the Kobayashi score includes seven variables and has been widely used in Japanese populations (11, 31). While these scoring systems show good sensitivity in Asian populations, their performance may vary in other regions (2). For patients resistant to initial IVIG therapy, second-line treatments are crucial. These may include corticosteroids, infliximab, or cyclosporine. Several studies in our review [e.g., Wu et al. (9); Cui et al. (4)] mentioned the use of adjunctive treatments, particularly corticosteroids, in refractory cases. According to AHA guidelines, early intensification of therapy in high-risk cases is recommended to reduce coronary complications (32).

The integration of such predictive models into clinical practice could enhance the precision of treatment plans and improve patient outcomes. The pathogenesis of KD and the mechanisms underlying IVIG resistance remain areas of active investigation. Further research into the molecular mechanisms driving IVIG resistance could uncover new therapeutic targets and improve the effectiveness of existing treatments. For instance, studies have suggested that genetic factors may influence an individual's response to IVIG, highlighting the potential for genetic screening in the future (33).

In addition to clinical and molecular research, epidemiological studies are essential to understand the broader impact of IVIG resistance in KD. Variations in resistance prevalence across different populations and regions suggest that environmental and genetic factors may play a role. This meta-analysis provides a global perspective on IVIG resistance, emphasizing the need for international collaboration in KD research.

In conclusion, this systematic review and meta-analysis have elucidated the prevalence and significant risk factors associated with IVIG resistance in KD. These findings emphasize the critical need for early identification and personalized treatment approaches to mitigate the risk of severe cardiac complications. Future research should continue to explore the molecular and genetic underpinnings of IVIG resistance, ultimately aiming to enhance therapeutic strategies and improve patient outcomes in KD.

## Limitations

This review is subject to limitations inherent in meta-analyses, including publication bias and variability in study methodologies. Additionally, the heterogeneity among populations studied may affect the generalizability of findings.

## Conclusion

The prevalence of IVIG resistance in Kawasaki disease remains a critical concern, affecting treatment outcomes and long-term cardiovascular health. Future research should focus on refining predictive scoring systems and exploring alternative treatment options for resistant cases.

## Data Availability

The original contributions presented in the study are included in the article/Supplementary Material, further inquiries can be directed to the corresponding author.
